# Direct comparison of serine restriction on the viability of normal and cancer cells demonstrates that methionine restriction is more cancer specific

**DOI:** 10.3389/fonc.2026.1931323

**Published:** 2026-09-01

**Authors:** Yuta Miyashi, Tomoyuki Ishiguro, Qinghong Han, Shukuan Li, Byung Mo Kang, Michael Bouvet, Yasunori Tome, Kotaro Nishida, Robert M. Hoffman

**Affiliations:** 1 AntiCancer, Inc., San Diego, CA, United States; 2 Department of Surgery, University of California, San Diego, San Diego, United States; 3 Department of Orthopedic Surgery, Graduate School of Medicine, University of the Ryukyus, Okinawa, Japan

**Keywords:** cancer, cancer cells, co-culture, glycine, Hoffman effect, methionine restriction, normal fibroblasts, serine

## Abstract

**Background/aim:**

Many studies in the present century have stated that restriction of serine is a cancer-specific vulnerability. The present study aimed to determine whether restriction of serine, compared to restriction of methionine, distinguishes cancer and normal cells.

**Materials and methods:**

143B-RFP osteosarcoma cells, HT1080-RFP fibrosarcoma cells, HCT116-GFP colon-cancer cells, and Hs27 normal fibroblasts were used in the present study. All cells are of human origin. Cancer and normal cells were cultured in RPMI-1640 medium without serine and glycine for serine/glycine restriction, and in Dulbecco’s modified Eagle’s medium without methionine for methionine restriction, each supplemented with 10% dialyzed fetal bovine serum. Cancer and normal cells were cultured in 96-well plates at 2 × 10^3^ cells/well. Serine, glycine, and methionine were added back as controls. Cell viability was measured with the WST-8 cell-viability reagent to establish dose-response curves for serine (with or without glycine) and for methionine in cancer and normal cells. Each cancer cell line was also co-cultured with Hs27 normal fibroblasts in 12-well plates. Each cell type was evaluated by phase-contrast and GFP/RFP fluorescence microscopy to determine the effects of serine/glycine restriction or methionine restriction.

**Results:**

Serine restriction alone did not distinguish cancer and normal cells. Both 143B osteosarcoma and Hs27 normal fibroblasts maintained approximately 100% viability without serine in glycine-containing medium. The HT1080-RFP and HCT116-GFP cancer cells lost approximately 40% of their viability without serine and showed increasing viability with increasing serine concentration. When both serine and glycine were restricted, Hs27 fibroblasts lost approximately 20% viability, whereas all cancer cells lost about 40% viability. Neither serine restriction nor serine/glycine restriction could lower the viability of cancer cells to 50% of control. In co-culture, the removal of serine and glycine still left many cancer cells viable. In contrast, methionine removal caused a much greater reduction in cancer-cell viability than removal of serine/glycine. Hs27 normal fibroblasts survived well under both serine/glycine and methionine restriction.

**Conclusion:**

Serine restriction and serine/glycine restriction are not as cancer-specific as methionine restriction.

## Introduction

Many studies in the current century have claimed that serine (Ser) restriction is a cancer-specific vulnerability. For example, in a study by Vander Heiden et al. (1), serine metabolism was proposed as a target for cancer therapy, since serine was said to be necessary for the specific needs of cancer cells. Vousden’s laboratory reported that cancer cells rapidly utilize exogenous serine (2). They also reported that serine deprivation activated the serine synthesis pathway (SSP). Another study reported substantial *de novo* serine biosynthesis in cancer (3). A subsequent study showed that dietary restriction of serine and glycine increased survival in transgenic mice with intestinal cancer due to inactivation of the *adenomatous polyposis coli* tumor-suppressor gene (4). Another study showed that inhibiting serine synthesis, combined with dietary restriction of Ser and glycine (Gly), slowed tumor growth (5). Another study reported that serine is vital for tumorigenesis and that cancer cells rely on serine uptake to meet increased biosynthetic demands (6).

Serine can be supplied to cancer cells through the SSP or taken up by the cell from the extracellular environment. However, it should be noted that cancer-cell lines differ in their response to serine deprivation and inhibition of phosphoglycerate dehydrogenase (PHGDH), which catalyzes the first reaction in the SSP. Some cancer-cell lines can be completely satisfied by the SSP, and others must consume large amounts of extracellular serine in order to be viable. Cancer cells can also adapt to serine starvation by increasing expressions of enzymes in the SSP. For example, oncogenes such as KRAS and MYC can increase expression of the enzymes that make the cancer cells more resistant to serine starvation (5). Loss of the *P53* gene can affect the SSP. Cancer cells, for example colon-cancer cell lines, can differ greatly from one another in their response to serine/glycine restriction or PHGDH inhibition (5).

However, the previous reports described above did not study cancer and normal cells under the same controlled conditions to determine the cancer-specific vulnerability to serine or serine/glycine restriction.

Methionine (Met) addiction, termed the Hoffman effect, is a fundamental and general hallmark that is specific to cancer. Normal cells are not addicted to Met. Studies since 1959 have shown that targeting Met by diet, a low-Met or Met-depleted cell culture medium, or methioninase treatment specifically inhibits cancer cells. Met addiction is due, at least in part, to elevated and aberrant transmethylation reactions in cancer cells (7–13).

The present study directly compared the serine/glycine requirement of cancer and normal cells with the methionine requirements of cancer and normal cells.

## Materials and methods

### Cell culture

The following human cancer cell types were used in the present study: HCT116 human colon-cancer cell line, the HT1080 human fibrosarcoma cell line, the 143B human osteosarcoma cell line, and Hs27 normal human fibroblaasts. All cell lines were obtained from the American Type Culture Collection (Manassas, VA, USA). Green fluorescent protein (GFP)-expressing HCT116 cells and red fluorescent protein (RFP)-expressing 143B and HT1080 cells were established at AntiCancer Inc. as described elsewhere (14, 15). The cells were maintained in Dulbecco’s modified Eagle’s medium (DMEM) supplemented with 10% fetal bovine serum and 1% penicillin/streptomycin (Thermo Fisher Scientific, Waltham, MA, USA) in an incubator at 37 °C with 5% CO_2_. These cell lines were randomly chosen.

### Serine dose-response with or without glycine of cancer and normal cells

Each cell line was seeded (2.0 × 10^3^ cells/well) in a 96-well plate with normal DMEM (100 µl/well) and incubated at 37 °C overnight. Ser/Gly-restricted medium was prepared with RPMI-1640 medium without glucose, Gly, and Ser (Teknova, Cat. No. R9660, Hollister, CA, USA) supplemented with only 20 mM glucose, 10% dialyzed fetal bovine serum, and 1% penicillin/streptomycin. Gly-containing medium was prepared by supplementing the medium described above with 250 µM Gly. Each cell line was treated with Gly-restricted or Gly-containing medium containing different concentrations of L-serine at 37 °C for 96 h, as follows: 0 µM, 2 µM, 4 µM, 8 µM, 16 µM, 32 µM, 64 µM, 128 µM, and 256 µM. After the treatment period, the WST-8 viability reagent (10 µl, Dojindo Laboratories, Kumamoto, Japan) was added to each well, and absorbance at 450 nm was measured after 1 h. Dose-response curves were fitted using a four-parameter logistic model in GraphPad Prism (version 10.6.1; GraphPad Software, San Diego, CA, USA). Experiments were performed in triplicate.

### Co-culture of cancer and normal cells to determine vulnerability to serine/glycine and methionine depletion

Each cancer cell line (5.0 × 10^4^ cells) was seeded into 12-well plates together with Hs27 normal fibroblasts (5.0 × 10^4^ cells). The day after seeding, following a wash with phosphate-buffered saline, the medium in each well was replaced with one of the following: Complete medium (MET^+^SER^+^, DMEM/F-12, GlutaMAX™ supplement, Cat. No. 10565018, supplemented with 10% fetal bovine serum and 1% penicillin/streptomycin, Thermo Fisher), Met-restricted medium [MET^-^SER^+^, DMEM, high glucose, no glutamine, no methionine, no cystine, Cat. No. 21013024, supplemented with 150 µM L-cystine 2HCl (300 µM cysteine), 4 mM of glutamine, 10% dialyzed fetal bovine serum, and 1% penicillin/streptomycin, Thermo Fisher Scientific, Waltham, MA, USA], or Ser- and Gly-restricted medium [MET^+^SER^-^GLY^-^, RPMI-1640 medium without glucose, Gly, and Ser, Teknova, Cat. No. R9660, supplemented with 20 mM glucose, 10% dialyzed fetal bovine serum, and 1% penicillin/streptomycin, Thermo Fisher Scientific].

### Imaging

Four days after the medium was replaced, the wells with co-cultures of cancer and normal cells were washed twice with phosphate-buffered saline. Phase-contrast and fluorescence microscopy images (for GFP or RFP fluorescence of cancer cells and normal cells) were acquired with an Olympus IX71 microscope (Olympus Corp., Tokyo, Japan).

## Results

### Effect of serine restriction on cancer and normal cells

**Figure 1:**
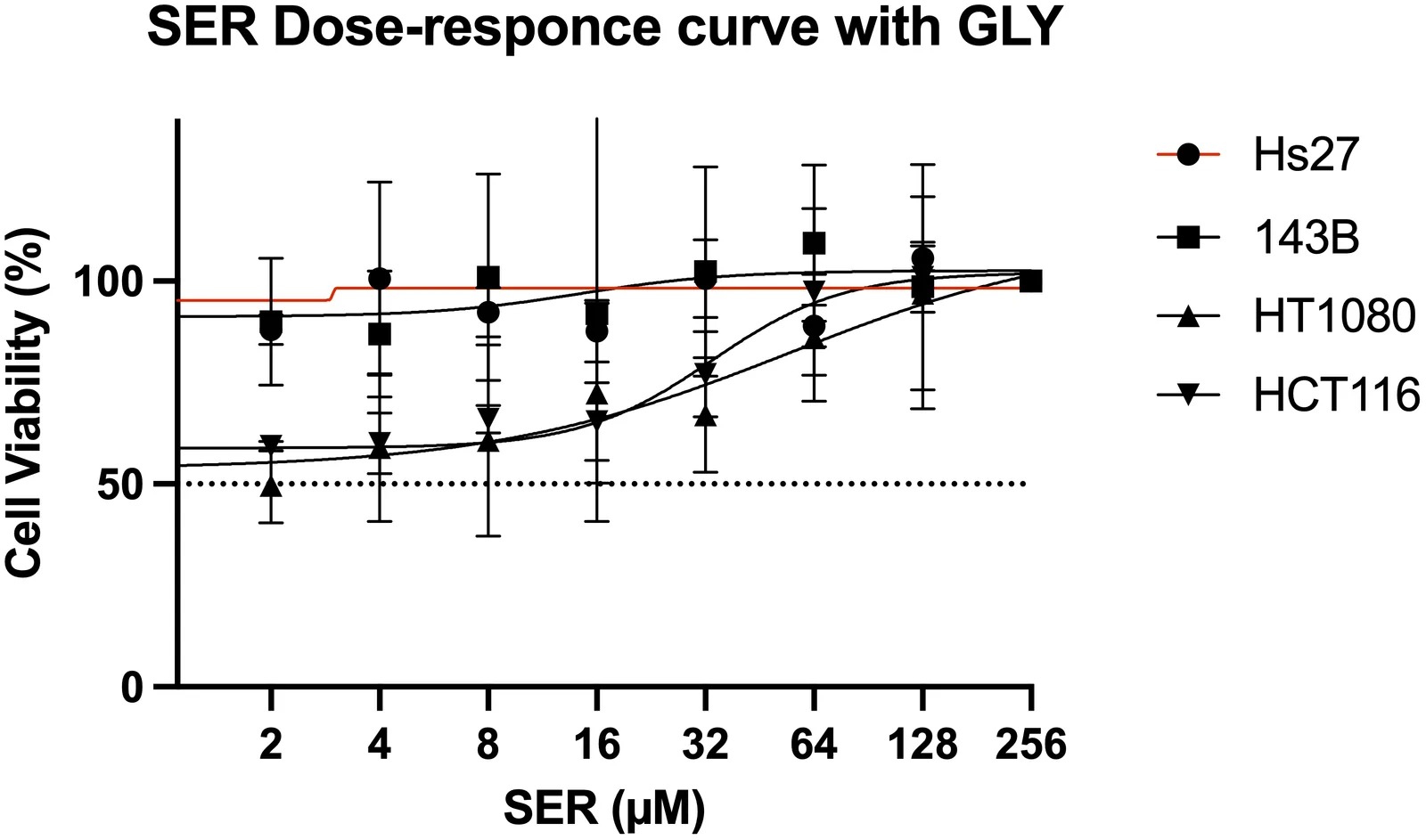
Dose-response curves of serine for each cancer and normal cell line in glycine-containing medium. Both 143B osteosarcoma and Hs27 normal fibroblasts maintained 100% viability even without serine (Ser) in glycine (Gly)-containing medium. The HT1080-RFP and HCT116-GFP cells lost approximately 40% of their viability without serine and showed increasing viability with increasing Ser, starting at 8 μM. HCT116 colon-cancer cells and Hs27 normal fibroblasts showed similar viability at 128 µM serine. Please see Materials and Methods for details.

Both 143B osteosarcoma cells and Hs27 normal fibroblasts maintained approximately 100% viability in serine-free, glycine-containing medium, indicating that normal cells and osteosarcoma cells synthesize sufficient serine for growth and that serine restriction had no effect on the cancer or normal cells. The HT1080-RFP and HCT116-GFP cells lost approximately 40%–50% of their viability without serine in glycine-containing medium and showed increasing viability with increasing serine, starting at 8 μM. HCT116 colon-cancer cells and Hs27 normal fibroblasts showed similar viability at 128 μM serine. Serine restriction in glycine-containing medium did not reduce the viability of either cancer or normal cells by more than 50% (**Figure 1**).

### Effect of combined serine and glycine restriction on cancer and normal cells

**Figure 2:**
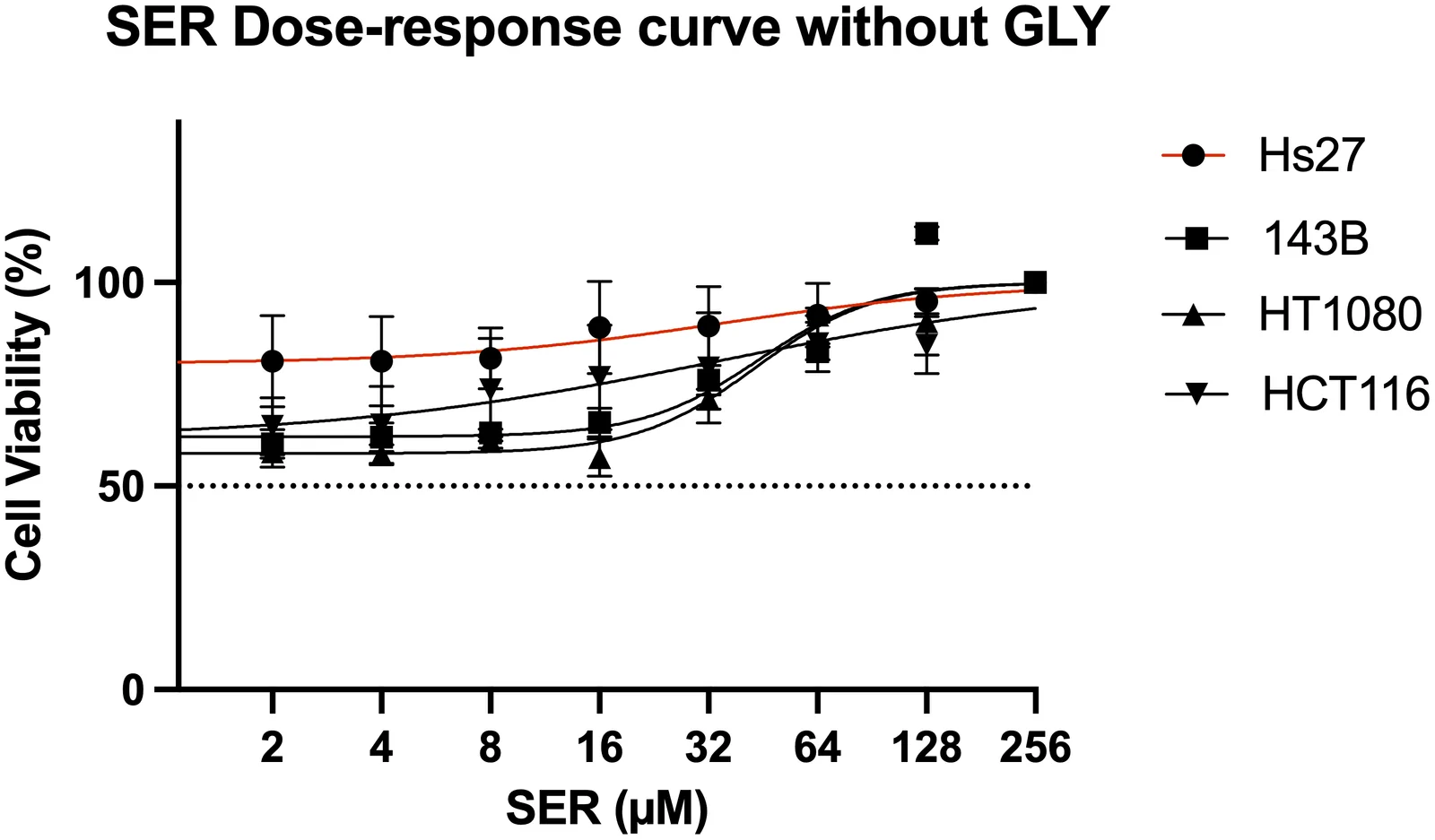
Dose-response curves of serine for each cancer and normal cell line in glycine-restricted medium. When both serine and glycine were restricted, normal fibroblasts lost about 20% viability, and cancer cells lost about 40% viability. Viability increased in cancer and normal cells with increasing serine concentration and was similar in the two cell types at approximately 64 μM Ser. Please see Materials and Methods for details.

When both serine and glycine were restricted, normal fibroblasts lost about 20% viability, and cancer cells lost about 40% viability. Viability increased in cancer and normal cells with increasing serine concentration and was similar in the two cell types at approximately 64 μM serine. Combined serine and glycine restriction did not reduce the viability of either cancer or normal cells by more than 50% (**Figure 2**).

### Effect of combined serine and glycine restriction compared to methionine restriction on co-cultured cancer and normal cells

**Figure 3:**
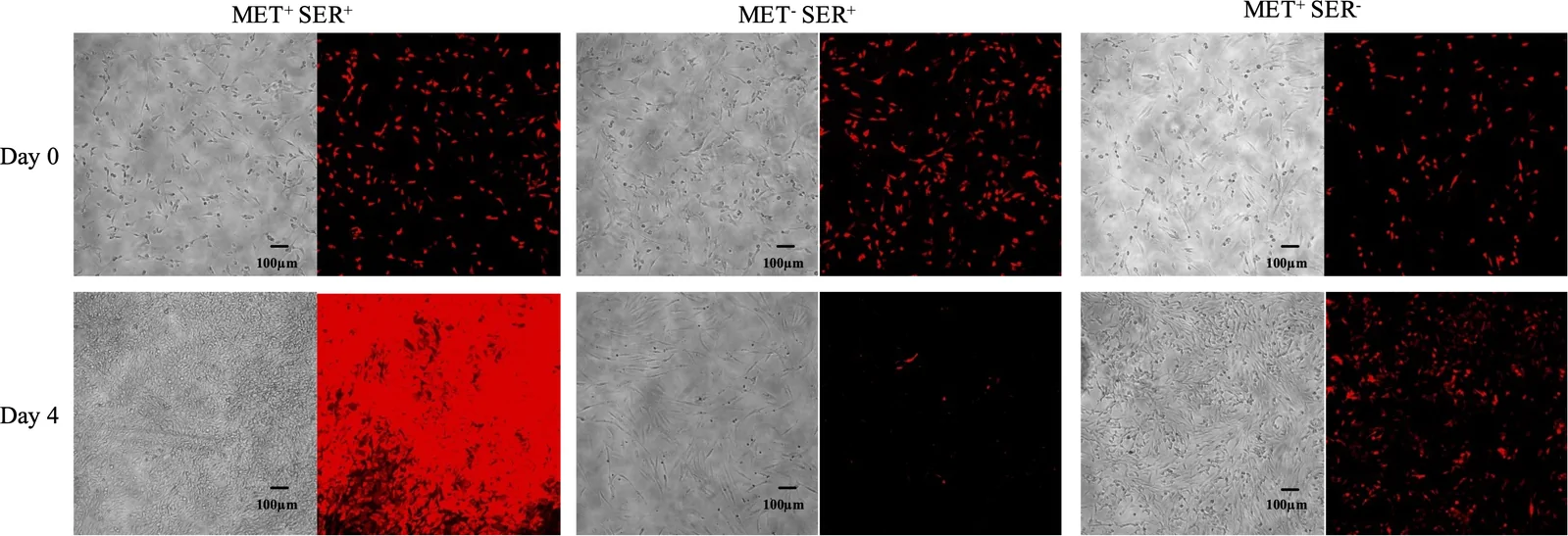
Co-culture of 143B-RFP osteosarcoma cells and Hs27 normal fibroblasts to determine cancer-specific vulnerability to serine/glycine or methionine restriction. In the methionine- and serine/glycine-containing (MET^+^SER^+^) medium, normal and cancer cells grew very well, with the cancer cells dominating the culture. In the methionine-restricted, serine/glycine-containing (MET^−^SER^+^) medium, the Hs27 normal fibroblasts were alive and healthy at day 4. In contrast, the 143B osteosarcoma cells were not viable at day 4, as visualized by phase-contrast microscopy or by RFP fluorescence. 143B osteosarcoma cells were viable in the methionine-containing, serine/glycine-restricted (MET^+^SER^−^) medium by day 4. Representative images are presented. Please see Materials and Methods for details.

**Figure 4:**
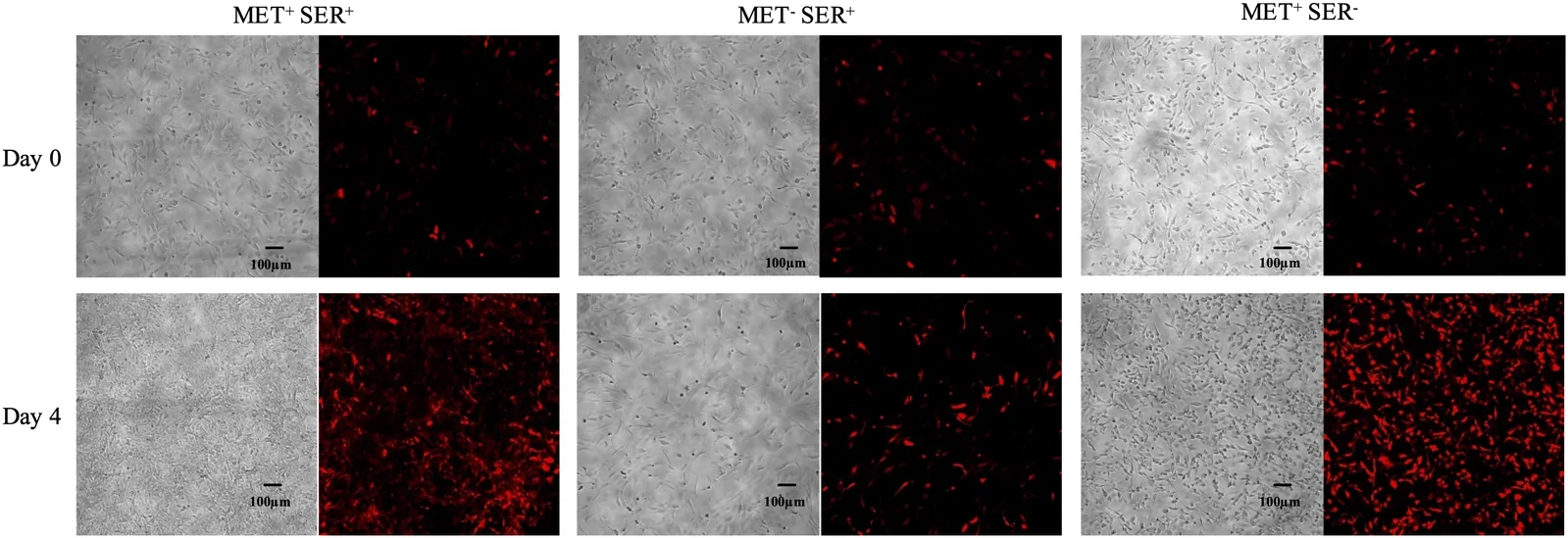
Co-culture of HT1080-RFP fibrosarcoma cells and Hs27 normal fibroblasts to determine cancer-specific vulnerability to serine/glycine or methionine restriction. In the methionine- and serine/glycine-containing (MET^+^SER^+^) medium, normal and cancer cells grew very well, with the cancer cells dominating the culture. In the methionine-restricted, serine/glycine-containing (MET^−^SER^+^) medium, the Hs27 normal fibroblasts were alive and healthy at day 4. In contrast, the HT1080 fibrosarcoma cells were not viable at day 4, as visualized by phase-contrast microscopy or by RFP fluorescence. HT1080 fibrosarcoma cells were viable in the methionine-containing, serine/glycine-restricted (MET^+^SER^−^) medium by day 4. Representative images are presented. Please see Materials and Methods for details.

**Figure 5:**
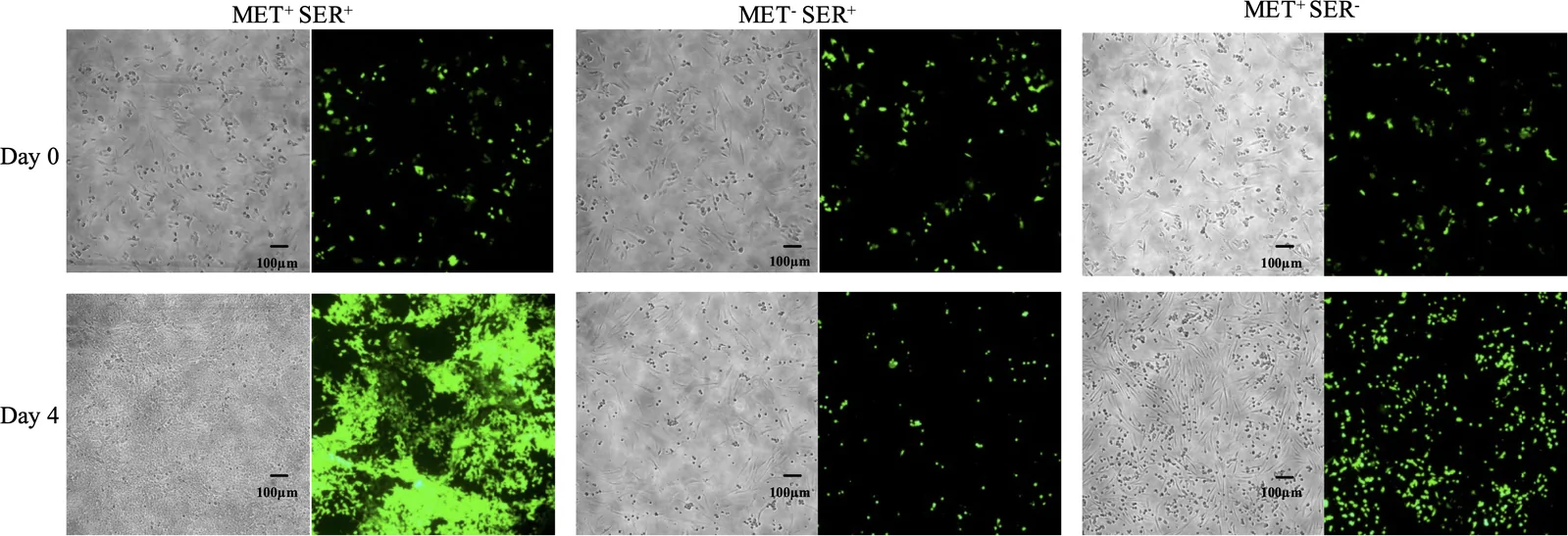
Co-culture of HCT116-GFP colon-cancer cells and Hs27 normal fibroblasts to determine cancer-specific vulnerability to serine/glycine restriction or methionine restriction. In the methionine- and serine/glycine-containing (MET^+^SER^+^) medium, normal and cancer cells grew very well, with the cancer cells dominating the culture. In the methionine-restricted, serine/glycine-containing (MET^−^SER^+^) medium, the Hs27 normal fibroblasts were alive and healthy at day 4. In contrast, the HCT116 colon-cancer cells were not viable at day 4, as visualized by phase-contrast microscopy or by GFP fluorescence. The HCT116 colon-cancer cells were viable in the methionine-containing, serine/glycine-restricted (MET^+^SER^−^) medium by day 4. Representative images are presented. Please see Materials and Methods for details.

In the co-culture experiments, removal of both serine and glycine still left many cancer and normal cells viable, as determined by their GFP/RFP fluorescence and phase-contrast microscopy, which could readily distinguish the rounded cancer cells from the highly elongated normal fibroblasts. In contrast, removal of methionine led to a greater reduction in cancer-cell viability than removal of serine and glycine. There was significant survival of normal Hs27 fibroblasts in the absence of either serine and glycine or methionine. Representative images are presented in **Figures 3**–**5**.

## Discussion

The comparison of cancer-cell and normal-cell variability used two pairs of media: serine/glycine-containing versus serine- or serine/glycine-restricted media, and methionine-containing versus methionine-restricted media. The present results show that serine restriction, with or without glycine restriction, cannot reduce the viability of any of the cancer cell lines tested or normal fibroblasts below 50% (**Figure 1**). The viability of 143B osteosarcoma cells was not affected by the removal of serine in the presence of glycine, similar to the effect on normal cells. The removal of both serine and glycine had a greater effect on cancer-cell viability but still did not reduce the viability of any cancer or normal cells below 50% (**Figure 2**). Neither the removal of serine alone nor the removal of serine plus glycine could reduce the viability of cancer and normal cells by more than 50%.

Both normal and cancer cells appear to produce sufficient serine to meet most of their needs, suggesting that serine or serine/glycine restriction has limited potential for cancer treatment.

Similar results were observed in the co-culture of normal and cancer cells, where removal of serine/glycine left many cancer cells and normal cells being viable (**Figures 3**–**5**). In contrast, removal of methionine had little effect on normal-cell viability but greatly reduced cancer-cell viability. Our previous co-culture studies also showed that methionine restriction reduced viability of and killed many cancer cells, while normal cells remained viable (16–20). The cancer cells were clearly marked by their GFP or RFP fluorescence and had a very different morphology from the elongated fibroblasts, making it easy to distinguish cancer cells from normal cells. The results shown in **Figures 3**–**5** are semi-quantitative; cancer cells and normal cells responded similarly to serine/glycine restriction but differently to methionine restriction. One limitation of the study is that a single normal human fibroblast, HS27, was used as the representative of normal cells. Future studies will use tissue-specific normal cells.

Previous studies asserting that serine or serine/glycine is a promising target for cancer therapy did not perform controlled comparisons with normal cells (1–6), as in the present study. A study by Baksh showed that what were termed “oncogenic” epidermal stem cells were serine auxotrophs. Their significance to the serine requirements of cancer itself is not clear (21). Recently, nano-delivery targeting serine biosynthesis to induce serine auxotrophy has been proposed (22). Colon cancer in a genetically engineered APC mouse model and lymphoma in a c-Myc-driven mouse model were inhibited by serine restriction (4).

The main result of the present study demonstrates that serine or serine/glycine restriction cannot sufficiently inhibit cancer-cell viability and is therefore not a promising therapeutic strategy. In addition, serine or serine/glycine restriction did not distinguish between cancer cells and normal cells. In contrast, the present study and studies conducted over the past nearly 70 years have shown that methionine restriction is a cancer-specific vulnerability (7–13, 16–20).

A limitation of the present study is the use of a single fibroblasts cell strain (Hs27) as the normal control. However, normal fibroblasts are among the most common cell types in the body, and they have been used as a normal control in numerous cancer research studies, including our own, for over 50 years (9). Future studies will also use tissue-specific normal cells.

In conclusion, the present results suggest that, compared to methionine restriction, serine/glycine restriction is not a cancer-specific vulnerability and is not a promising therapeutic strategy for cancer.

## Data Availability

The original contributions presented in the study are included in the article/supplementary material. Further inquiries can be directed to the corresponding authors.

## In Memoriam

This paper is dedicated to the memory of A. R. Moossa, MD; Professor Philip Miles; Sun Lee, MD; Richard W. Erbe, MD; Professor Milton Plesur; Professor Gordon H. Sato; Professor Li Jiaxi; Masaki Kitajima, MD; Shigeo Yagi, Ph.D.; Jack Geller, MD; Joseph R. Bertino, MD; J. A. R. Mead, PhD; Eugene P. Frenkel, MD; John Mendelsohn, MD; Professor I. J. Fidler; Professor Lev Bergelson; Professor Sheldon Penman; Professor John R. Raper; Professor Peter H. Duesberg; Professor J. D. Watson; and Joseph Leighton, MD. May their memory be a blessing.
